# Letter of Dr. Nunez to Dr. Bœnninghausen*Written probably in 1846. Dr. Nunez was the ablest homœopathist who ever practiced in Spain.—Eds.

**Published:** 1889-08

**Authors:** 


### LETTER OF DR. NUNEZ TO DR. BŒNNINGHAUSEN.*

It is now a year since I last wrote you. Since then my views and my practice have undergone great changes. You are right, my friend; the recently introduced highest potencies are a real progress in Homœopathy, and I believe that this progress would be still more considerable if we instituted our provings upon the healthy body with the 200th potencies. You were right when you assured me that the results which we can obtain with the highest potencies, are truly marvelous. I am even disposed to believe that the potencies beyond the 300th are more efficacious than the 200th. Of the 300th, I have seen marked exacebations.

In a former communication I told you of a marked exacerbation occasioned by Calc.$^{200}$, in a case of chronic myelitis, of which the dean of the faculty of Barcelona was suffering. Since then I have seen a still more marked exacerbation from *Calc.*$^{700}$ in a case of acne rosacea. With doses of *Sepia*$^{1200}$ I have effected a completed cure of chronic constipation of forty years' standing in a lady of seventy-six years, which had become so inveterate that the patient never had a natural evacuation, and had to use mechanical application whenever she wished to have relief; the rectum seemed to be entirely inactive. Since then I have cured several other cases of chronic constipation with *Sepia*$^{1200}$, and have never failed in any case of that kind. *Arsenic*$^{1200}$ has cured spitting of blood, accompanied with suppression of the menses, obstinate constipation, burning pain in the stomach and between the scapulæ, all these symptoms of four years' standing;

* Written probably in 1846. Dr. Nunez was the ablest homœopathist who ever practiced in Spain.—Eds.

one dose was sufficient to remove them. *Nux*$^{200}$ and *Sulphur*$^{1200}$ in alternation have cured two cases of tuberculous phthisis at the stage of softening. One single dose of *Ledum*$^{300}$ has cured a case of sciatica which had been treated allopathically for six months without the least benefit.* One dose of *Sulphur*$^{1200}$ has cured a diarrhœa of eighteen months' standing, attended with phthisicky symptoms; the diarrhœa had been occasioned by the abuse of Copaiva in a case of blennorrhœa from the urethra. One dose of *Cantharides*$^{200}$ was sufficient to cure a chronic catarrh of the bladder with hæmaturia and spasmodic closing of the neck of the bladder. Three doses of *Silicea*$^{1000}$ have cured a swelling of the size of a plum in a scrofulous child of eleven years, occasioned by the closing of an issue; the swelling was seated between the fifth and sixth ribs on the right side below the nipple, and had been treated with Hydriodate of Potash and poultices, by which the swelling had become larger. One dose of *Crocus*$^{200}$ arrested at once a violent hemorrhage from the uterus. *Veratum*$^{300}$, two doses, has cured a case of diabetes with violent thirst, obliging the patient to hold a moist sponge in his mouth constantly. Three doses of *Natrum mur.*$^{300}$ have cured two cases of chronic gonorrhœa, one of fourteen months and the other of three years' standing.

Aggravations occasioned by the 200th dynamizations are sometimes very violent and obstinate. I gave *Natr. mur.*$^{200}$ for a chronic gleet, and a complete retention of urine was occasioned by it, which yielded to *Conium.*† In another case of that kind, I gave *Sulphur*$^{200}$, four days in succession; on the fifth a frightful inflammation of the bladder set in. One dose of *Calcarea*$^{700}$ occasioned a violent congestion of blood to the head and heart, with suffocative fits and loss of consciousness. To a nervous lady, who had been in the habit of being bled, I gave one pellet of *Arsenic*$^{300}$; one hour after taking it, violent retching set in, and half an hour after the menses made their appearance, eighteen days before the regular period. This lady had always been regular, and had never had an attack of retching.

I have founded a Homœopathic Society in Madrid, consisting of twenty-four members. The President of the State Ministry

---

* With Colocynthis$^{200}$ in water, one dose a day, I have cured a case of nervous sciatica of nine months' standing, in a military man who had been treated all this time with the usual allopathic means without benefit. I cured him in six days.—STAPF.

† A robust female, of thirty years, took Natr. mur.$^{400}$ for a chronic leucorrhœa, after which it became excessively violent and corrosive. After the aggravation had lasted four days, the leucorrhœa disappeared entirely and permanently.—STAPF.

has appointed me his physician; and it has been determined that lectures on Homœopathy shall be given in the University. The lectures will comence on the 1st of January. Send me your Therapeutic Pocket-Book as soon as it is out.

